# Determinants of an autism spectrum disorder diagnosis in childhood and adolescence: Evidence from the UK Millennium Cohort Study

**DOI:** 10.1177/1362361320913671

**Published:** 2020-05-05

**Authors:** Mariko Hosozawa, Amanda Sacker, William Mandy, Emily Midouhas, Eirini Flouri, Noriko Cable

**Affiliations:** 1 University College London, UK; 2 Juntendo University, Japan

**Keywords:** adolescents, autism spectrum disorder, diagnosis, Millennium Cohort Study

## Abstract

This study aimed to identify determinants of a late autism spectrum disorder diagnosis, including diagnoses made ‘very late’ (i.e., in adolescence), using the Millennium Cohort Study, a nationally representative population-based cohort in the United Kingdom. Children diagnosed with autism spectrum disorder by age 14 (N = 581) were included and grouped by the parent-reported timing of diagnosis: before school (up to age 5), during primary school (age 5–11) and during secondary school (age 11–14). Predictors of diagnostic timing, at the child, family and school levels, were investigated using multinomial logistic regression. Most (79%) children with autism spectrum disorder were diagnosed after school entry, and 28% were not diagnosed until secondary school. Among those not diagnosed until secondary school, 75% had been identified at age 5 years by a parent and/or teacher as having socio-behavioural difficulties. Being diagnosed after starting school was predicted by living in poverty (adjusted relative risk ratio: primary = 1.90, 95% confidence interval: 1.03–3.53; secondary = 2.15, 1.05–4.42) and/or having no initial parental concerns (primary = 0.32, 0.15–0.70; secondary = 0.19, 0.09–0.43). Having typical-range intelligence also predicted diagnosis during secondary school. The result indicates that those without cognitive delays and poorer children were at risk of ‘very late’ (i.e. adolescent) diagnosis. Strategies to promote earlier identification, targeting age at primary school entry, could help those more likely to be diagnosed late.

Lay abstract

Despite policy emphasis on early identification, many children with autism spectrum disorder are diagnosed late, with some being diagnosed as late as in adolescence. However, evidence on what determines the timing of autism spectrum disorder diagnosis including children diagnosed in adolescence is lacking. Understanding these determinants, particularly in those diagnosed later than is ideal, can inform the development of effective strategies to improve earlier identification of autism spectrum disorder. This study used a nationally representative population-based cohort in the United Kingdom to explore child, family and school level predictors of timing of autism spectrum disorder diagnosis. In the United Kingdom, 79% of the children with autism spectrum disorder were diagnosed after entering primary school and 28% during secondary school. Among those not diagnosed until secondary school, 75% had shown social difficulties noticed by parents and/or teachers at age 5 years. The results suggest that healthcare providers should be aware that, even for universal systems of care, those living in poverty and having higher intelligence are most likely to miss out on a timely diagnosis. Strategies to promote earlier identification among school-aged children, including targeting primary school entry age (i.e. around age 5) and that encouraging referrals for a formal assessment at the first report of concerns over the child’s social development may benefit those children who would otherwise be diagnosed later.

Autism spectrum disorder (ASD) is a neurodevelopmental condition characterized by impairments in social relating, social communication, flexibility and sensory processing (American Psychiatric Association, 2013). People with ASD are at risk for significant social, emotional and economic difficulties across the lifespan, especially if their condition is undiagnosed and unsupported (Bargiela et al., 2016; Lai & Baron-Cohen, 2015). Early identification is essential to improve their prognosis through timely intervention (Dawson, 2008; Zuckerman et al., 2017) and adequate support at school and at home (Reed & Osborne, 2012; Ruiz Calzada et al., 2012). ASD can be reliably diagnosed by as early as 24 months of age (Corsello et al., 2013; Steiner et al., 2012), and current policy in many developed nations seeks to promote early identification of ASD (National Initiative for Autism: Screening and Assessment [NIASA], 2003; National Institute for Health and Care Excellence [NICE], 2011).

Despite efforts to promote timely ASD diagnoses, a recent study reported that age at diagnosis in the United Kingdom did not decrease between 2004 and 2014: nearly half of the children with ASD in their sample were diagnosed only after they had started school (Brett et al., 2016). Also, the mean age of ASD diagnosis in the United States was 53 months in 2006 and 52 months in 2014, showing that there has been little increase in early identification during the last decade (Baio et al., 2018). These findings suggest that there is an ongoing need to develop strategies that promote earlier identification of ASD.

Understanding factors that determine the timing of ASD diagnosis, particularly in those diagnosed later than is ideal (i.e. after they have started school at around age 5), can inform the development of effective strategies to improve earlier identification of ASD. Previous studies have shown that receiving a late ASD diagnosis is associated both with clinical factors such as higher cognitive ability (Mazurek et al., 2014; Shattuck et al., 2009; Zwaigenbaum et al., 2019), milder autistic symptoms (Fountain et al., 2011; Mandell et al., 2005; Mazurek et al., 2014; Sheldrick et al., 2017; Zwaigenbaum et al., 2019), diagnostic overshadowing (Miodovnik et al., 2015) and with socio-demographic factors including sex, socio-economic status and access to healthcare (Bickel et al., 2015; Brett et al., 2016; Daniels & Mandell, 2014; Fountain et al., 2011; Mandell et al., 2005; Mazurek et al., 2014; Shattuck et al., 2009; Sheldrick et al., 2017; Williams et al., 2008; Zwaigenbaum et al., 2019). It should be acknowledged, however, that findings about socio-demographic factors have been inconsistent, likely due to methodological differences and variation between countries in their healthcare systems (Daniels & Mandell, 2014).

Nevertheless, there are currently important gaps in our understanding of late ASD diagnosis that constrain efforts to address this problem. First, most studies have focused on those in primary education, meaning that we know little of what factors predict very late diagnoses, that is, those given in adolescence (Fountain et al., 2011; Shattuck et al., 2009; Williams et al., 2008; Zwaigenbaum et al., 2019). Second, much of the evidence in this area is based on data from clinic-referred samples, limiting its generalizability (Bickel et al., 2015; Brett et al., 2016; Mandell et al., 2005; Mazurek et al., 2014). Third, most of the studies were conducted in the United States where the healthcare system is not universal (Bickel et al., 2015; Daniels & Mandell, 2014; Fountain et al., 2011; Mandell et al., 2005; Mazurek et al., 2014; Shattuck et al., 2009; Sheldrick et al., 2017). For countries with a universal healthcare system, it is important for policymakers to understand whether socio-economic disparity plays a role in the timing of diagnosis in that system. Finally, longitudinal studies are lacking, limiting our understanding of ‘red flags’ for missed diagnosis – that is, early characteristics of children at risk of receiving a late diagnosis, such as early concerns of the parents and educators (Daniels & Mandell, 2014).

We investigated the child, family and teacher factors that predict the timing of diagnosis for ASD using the UK Millennium Cohort Study (MCS), an ongoing population-based birth cohort study of children born around the millennium. Particular emphasis was placed on identifying the determinants of timing of diagnosis in relation to key educational stages: (1) up to primary school (i.e. age 5 in the United Kingdom); (2) during primary school from age 5 to 11; or (3) during secondary school from age 11 to 14. Our aim was to yield information that can help parents, clinicians, educational practitioners and policymakers improve earlier identification of ASD.

## Methods

### Study population

The MCS is a population-representative birth cohort study in the United Kingdom, following the health and development of children from 19,243 families who were born in the United Kingdom between September 2000 and January 2002. Details are described elsewhere (Hansen, 2014). Of the 15,459 children who took part in MCS at age 5 (when information on ASD diagnosis was first collected), we excluded those without valid information on ASD diagnosis in all four available sweeps (i.e. age 5, 7, 11 and 14 sweeps, n = 28). Those whose parents reported a diagnosis of ASD by the age 14 sweep (n = 581) were included in this study. Data were obtained from the UK Data Archive (further information found at cls.ucl.ac.uk/cls-studies/millennium-cohort-study/). The MCS is approved by the UK National Health Service Research Ethics Committee and written consent was obtained from all participating parents at each survey. The use of anonymized data for academic purposes did not require additional ethical approval.

### Outcome: timing of ASD diagnosis

Parents were asked, ‘Has a doctor or other health professional ever told you that your child had Autism, Asperger’s Syndrome or other autistic spectrum disorder?’ when the child was approximately 5, 7, 11 and 14 years old. This question has been used to ascertain ASD diagnosis in other population-based studies. Any child whose parent responded ‘yes’ to this question at one of the time-points was identified as having an ASD diagnosis (Kogan et al., 2009; Miodovnik et al., 2015). A three-category variable was then derived, based on the UK school stage the child was in when the parent first reported an ASD diagnosis: (1) ‘before school’ if reporting an ASD diagnosis at the age 5 interview when the child was in preschool (nursery or reception class); (2) ‘during primary’ if at age 7 or 11 and when the child was in primary school; (3) ‘during secondary’ if at age 11 or 14 and when the child was in secondary school. Eight children whose age 5 interview took place in September (the first month in school Year 1) and whose parents reported a diagnosis were included in the ‘before school’ group on the assumption that the child had received a diagnosis before starting primary school.

### Explanatory variables

#### Child’s cognitive ability at age 5

The child’s cognitive ability was obtained from three subscales of the British Ability Scales II (BAS II) administered at age 5: the BAS II Naming Vocabulary Subscale indicative of the level of the spoken vocabulary, the BAS II Picture Similarity Subscale indicative of problem-solving abilities and the BAS II Pattern Construction Subscale indicative of spatial awareness (Elliott et al., 1997). A total score for each BAS II subscale that was adjusted for age and difficulty was standardized to have a mean of 50 with a standard deviation (SD) of 10 (Elliott et al., 1997). A total cognitive score was derived by taking the mean of the three subscales. Those scoring below 1 SD (<40) were identified as having a cognitive delay.

#### Parental concerns over the child’s socio-behavioural difficulties at age 5

Parents were asked about their concerns over the child’s overall social, emotional or behavioural difficulties at age 5 as follows: ‘Overall, do you think that your child has difficulties in one or more of the following areas: Emotions, concentration, behaviour, or being able to get on with other people?’ Those answering ‘yes’ to the question were categorized as having concerns about their child’s difficulties.

#### Teacher-evaluated social developmental delay at age 5

Teachers evaluated the child’s social development at age 5 using a questionnaire that mimicked the Foundation Stage Profile; teachers completed this questionnaire at the end of preschool, when children were aged between 4 and 5, to assess early learning goals in children (Johnson, 2008). This comprises 13 subscales that address six areas of development, with each subscale having nine questions (subscale scores range 0–9, with higher scores indicating more developed ability). This questionnaire is well validated. We used the social development subscale; however, there were some differences in the distribution of the responses for this variable between England, Wales, Scotland and Northern Ireland (Johnson, 2008). Therefore, in each country, we classified those scoring below 1 SD as having a social developmental delay at age 5.

### Covariates

The covariates below, all measured at age 5, were included in the study model: child’s sex, multiple birth indicator, highest parental educational attainment (attaining Advanced level which is a qualification required to enter university, or higher), relative income poverty of the household (indicated by the equivalized household income being less than 60% of the UK national median household income) and whether living in a highly health-deprived area (defined as living in the top 10% of health-deprived areas within the United Kingdom, based on the health deprivation and disability domain of the Index of Multiple Deprivation (IMD) 2004/2005).

### Statistical analyses

We applied multivariable multinomial logistic regression to examine the predictors of each diagnostic-age group by taking the ‘before school’ group as the reference category (adjusted relative risk ratios (aRRR) were given). To further capture the between-group differences aside from the reference group, differences in regression coefficients between ‘during primary’ and ‘during secondary’ groups were estimated and formally compared. We further conducted a post-estimation analysis to predict the relative effect of each factor on the outcome groups, using the Stata module ‘mimrgns’ (Klein, 2014). All analyses were adjusted for the multiple birth indicator and were weighted using survey and non-response weights to account for the MCS clustered sampling design and participant attrition. All analyses were conducted using Stata SE version 15 (StataCorp, College Station, TX).

### Sensitivity analysis

To test the robustness of the observed associations, we conducted the following three analyses: (1) we repeated the analysis by replacing the ‘parental concerns over the child’s socio-behavioural difficulties at age 5’ by indicators of parent-rated ‘social difficulties’ defined as scoring either above 1 SD on the peer subscale (higher scores indicate more peer problems) or below 1 SD on the prosocial subscale (lower scores indicate fewer prosocial problems) of the whole MCS population in the Strengths and Difficulties Questionnaire (Goodman, 1997) reported by the parent when the child was aged 5; (2) we repeated the analysis by excluding children who had reported receiving a diagnosis of attention-deficit/hyperactivity disorder (ADHD) prior to reporting receiving a diagnosis of ASD to explore whether preceding ADHD diagnosis would affect the results; and (3) we repeated the analysis by excluding 15 children in the ‘before school group’ whose parents reported losing their ASD diagnosis in the following sweep (information for change in diagnostic status was only available between the age 5 and 7 interviews).

### Missing data

Missing data on each variable ranged from 0.2% (on parental educational attainment) to 25.9% (on social development). We imputed missing cases using multiple imputation by chained equations using all the variables included in the analysis models and auxiliary variables to minimize data loss (Harel et al., 2018; White et al., 2011). To account for the different distribution of teacher-evaluated social development between England and the other UK countries, imputation was conducted separately for England and these countries using the same imputation model. Imputed data for each country were then merged into one dataset. Regression analyses were run across 25 imputed datasets and adjusted using Rubin’s rules (White et al., 2011). Imputed results were broadly similar to those obtained using observed samples (Supplemental Table S1) and therefore the former are presented here.

### Patient and public involvement

Participants of the MCS were not involved in setting the research question or the outcome measures, nor were they involved in the design or implementation of the study. No participants were asked to advise on the interpretation or writing up of the results. However, the results are disseminated to study participants through their dedicated website: https://childnc.net/.

## Results

### Descriptive characteristics of the children with ASD by the timing of diagnosis

The characteristics of the children with ASD in our sample by the timing of diagnosis are presented in Table 1. Of the 581 children diagnosed with ASD by age 14, only 21.4% (n = 126) were diagnosed before school and, notably, 27.9% (n = 155) were diagnosed after secondary school entry. Those without cognitive delay were more likely to be diagnosed after school entry. Low household income was least evident in the earliest diagnosed group compared to the other groups, whereas lower parental educational attainment was more prevalent in the earliest and the latest diagnosed groups (P_for quadratic trend_ = 0.004).

**Table 1.** Characteristics of children diagnosed with autism spectrum disorder (ASD) by the timing of diagnosis for ASD (N = 581).

	Multiply imputed sample (N = 581)			
	Before school (n = 126, 21.4%)	During primary school (n = 300, 50.7%)	During secondary school (n = 155, 27.9%)	Total ASD (N = 581)
	%	%	%	%
Sex of the child				
Male	80.6	76.9	73.3	76.7
Female	19.4	23.1	26.7	23.3
Cognitive ability^a^				
Within normal range	81.4	86.0	92.2	86.8
Below 1 SD	18.6	14.0	7.8	13.2
Parental highest education				
A-level^b^ or above	46.0	63.2	51.1	56.1
Below A-level^b^	54.0	36.8	48.9	43.9
Low household income^c^				
Yes	31.1	37.3	45.5	38.3
No	68.9	62.7	54.5	61.7
Neighbourhood health deprivation^d^				
Yes	7.5	10.6	15.2	11.2
No	92.5	89.4	84.8	88.8

Imputed and weighted percentages are shown.

a Cognitive delay defined as scoring 1 SD below average on subscales of British Ability Scales assessed at age 5.

b A-level is a qualification required to enter university.

c Below 60% of UK national median household income.

d Living in the top 10% of within-UK-country health-deprived areas (as measured using the health deprivation and disability domain of the Index of Multiple Deprivation 2004/2005).

**Figure 1.**
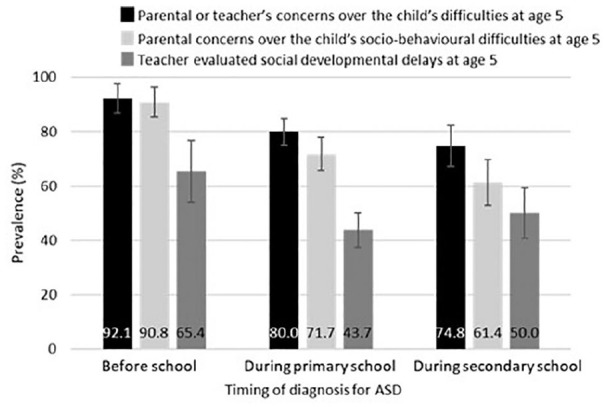
Prevalence of parental concerns over the child’s socio-behavioural difficulties (P_for linear trend_ < 0.001), teacher-evaluated social developmental delays (P_for quadratic trend_ = 0.005) and parental or teacher’s concerns over the child’s difficulties (P_for linear trend_ < 0.001) assessed at age 5 by the timing of diagnosis for ASD. Teacher’s concerns represent teacher evaluated social development delay. Imputed and weighted percentages are shown in the graph. Error bars show 95% CIs.

Figure 1 shows parent and teacher concerns over the child’s socio-behavioural difficulties at age 5. Prevalence of initial parental concerns over their child’s socio-behavioural difficulties declined in a trend along with the timing of diagnosis (P_for linear trend_ < 0.001). It is worth noting that half (50.0%) of those diagnosed during secondary school had been identified by their teachers at age 5 as having social difficulties, and 74.8% either by their parents or by their teachers at age 5 (Figure 1). Further detailed information on the descriptive characteristics of our study variables is available in Supplemental Table S1/S2.

### Identifying determinants of the timing of ASD diagnosis

Findings from the multivariable multinomial logistic regression analyses identified characteristics of the children with ASD and their parents that were independently associated with the timing of receiving the diagnosis (Table 2). In reference to the ‘before school’ group, membership of the ‘during primary’ or ‘during secondary’ groups was predicted by low household income (aRRR = 1.90, 95% confidence interval (CI) = 1.03–3.53; 2.15, 1.05–4.42, respectively) and not having initial parental concerns over the child’s difficulties (aRRR = 0.32, 0.15–0.70; 0.19, 0.09–0.43, respectively). Further comparison of regression coefficients between the ‘during primary’ and ‘during secondary’ school groups showed that not having cognitive delay and not having parental concerns were predictive of the ‘during secondary’ group compared to the ‘during primary’ group (Table 2).

**Table 2.** Adjusted relative risk ratio (95% confidence intervals) for determinants of the timing of diagnosis for autism spectrum disorder.

	Timing of diagnosis for autism spectrum disorder	
	During primary school^a^	During secondary school^a^
Female sex	1.02 (0.53–1.97)	1.23 (0.61–2.48)
Cognitive delay	1.12 (0.50–2.53)^b^	0.49 (0.19–1.30)^b^
Low parental education (below A-level)	0.42 (0.24–0.74)	0.68 (0.39–1.18)
Low household income	1.90 (1.03–3.53)	2.15 (1.05–4.42)
Having parental concerns over the child’s socio-behavioural difficulties at age 5	0.32 (0.15–0.70)^b^	0.19 (0.09–0.43)^b^
Teacher-evaluated social developmental delay	0.50 (0.27–0.90)	0.75 (0.39–1.44)
Neighbourhood health deprivation	1.65 (0.82–3.32)	1.90 (0.68–5.27)

a The Before school group is taken as reference.

b Significant difference between During primary school group and During secondary school group. All analyses adjusted for multiple birth indicator.

Post-estimation predicted probabilities confirmed that, given their other background characteristics as observed, children in the typical-range of cognitive ability were twice as likely – compared to those with cognitive delays – to be diagnosed during secondary school. Children whose parents had ‘concerns’ at age 5 were almost three times more likely to be diagnosed before school age whereas not having concerns increased the probability of being diagnosed in secondary school by approximately 50% (Table 3). The results of the sensitivity analysis showed similar results in general (Supplemental Tables S3–S5).

**Table 3.** Predicted probability (95% confidence interval) of the timing of diagnosis by characteristics of the children and their environment.

	Predicted probabilities for each group^a^		
	Before school	During primary school	During secondary school
Sex of the child			
Male	0.22 (0.18–0.26)	0.51 (0.46–0.57)	0.27 (0.23–0.31)
Female	0.20 (0.12–0.29)	0.49 (0.38–0.60)	0.31 (0.20–0.41)
Cognitive ability			
Within normal range	0.21 (0.17–0.26)	0.49 (0.44–0.55)	0.30 (0.25–0.34)
Below 1 SD	0.22 (0.12–0.32)	0.62 (0.50–0.74)	0.16 (0.07–0.25)
Parental highest education			
A-level or above	0.17 (0.12–0.21)	0.57 (0.51–0.64)	0.26 (0.20–0.31)
Below A-level	0.28 (0.21–0.34)	0.42 (0.34–0.50)	0.30 (0.23–0.37)
Low household income			
Yes	0.16 (0.10–0.22)	0.53 (0.46–0.61)	0.31 (0.24–0.38)
No	0.26 (0.20–0.31)	0.49 (0.43–0.55)	0.26 (0.20–0.31)
Having parental concerns over the child’s socio-behavioural difficulties at age 5			
Yes	0.25 (0.20–0.30)	0.51(0.45–0.57)	0.24 (0.19–0.28)
No	0.09 (0.04–0.14)	0.51 (0.42–0.60)	0.40 (0.31–0.49)
Teacher-evaluated social developmental delay			
Yes	0.25 (0.20–0.31)	0.44 (0.37–0.51)	0.30 (0.24–0.37)
No	0.17 (0.11–0.22)	0.58 (0.50–0.65)	0.26 (0.20–0.32)
Neighbourhood health deprivation			
Yes	0.15 (0.06–0.23)	0.53 (0.39–0.67)	0.33 (0.16–0.49)
No	0.22 (0.18–0.26)	0.50 (0.45–0.56)	0.27 (0.23–0.32)

SD: standard deviation.

a Post-estimation probabilities obtained from the parameters of the multivariate multinomial logistic analysis (Table 2). Observed values for each factor used in the estimation.

## Discussion

Using a prospective population-based cohort study of children in the United Kingdom, we examined the factors associated with the timing of diagnosis for ASD. Contrary to the stated goals of UK diagnostic policy (NIASA, 2003; NICE, 2011), most children with ASD in the United Kingdom were diagnosed after school entry; notably, 28% were not diagnosed until after starting secondary school. This is despite the fact that the majority (75%) of these very-late-diagnosed young people had been identified by a parent or teacher as having significant socio-behavioural problems at primary school entry. Living in a poor family and lack of parental concerns about socio-behavioural difficulties at age 5 were associated with being diagnosed after school entry. Post-estimation further revealed that not having cognitive delay was also associated with an increased chance of being diagnosed during secondary school compared to during primary school.

Consistent with recent literature (Brett et al., 2016; Sheldrick et al., 2017), many children were not diagnosed with ASD until reaching school age. However, in our study, the proportion of children diagnosed at school age (78.6%) was higher than in a prior UK report which was based on a clinically referred sample (51.1% in 2014; Brett et al., 2016). By contrast, we drew our study sample from a population-based birth cohort, suggesting that our findings may be more representative of current diagnostic practice. In addition, the increase in public awareness of ASD during our study period may have contributed to the increase in children who were referred and diagnosed for ASD at later ages (Hansen et al., 2015; Idring et al., 2015).

We, like others, found that those without cognitive delay (Mazurek et al., 2014; Shattuck et al., 2009; Zwaigenbaum et al., 2019) and those whose parents did not have concerns over their socio-behavioural difficulties were diagnosed later (Daniels & Mandell, 2014). Several potential explanations might underlie this result. First, it could be that those without cognitive delays also had milder forms of ASD symptoms and therefore were diagnosed later when their difficulties became apparent with age-related increasing social demands (Livingston & Happe, 2017; Mandy et al., 2018). Second, it may be that those without cognitive delays were better at compensating for social difficulties (i.e. camouflaging) also leading to later diagnosis (Hull et al., 2017). Third, not having cognitive delays could have made the parents or teachers overlook their concerns over the child’s social difficulties. Relatedly, diagnostic overshadowing could be a factor, whereby autistic characteristics could have been mistakenly attributed to a co-occurring conditions, such as ADHD, thereby delaying ASD diagnosis (Miodovnik et al., 2015). However, we directly investigated this possibility with respect to ADHD, and found no evidence for diagnostic overshadowing. The proportion of children diagnosed as ADHD by age 14 was lowest among groups in the ‘during secondary’ group, and excluding those with a preceding diagnosis of ADHD did not change the study’s main findings. It would be important in future research to include detailed information on healthcare usage (i.e. when the children were referred for consultation and/or clinical evaluation) and comorbid psychiatric diagnosis to understand which part of the diagnostic process to target to promote earlier diagnosis in school-aged children, particularly among those with typical-range cognitive ability.

Although lack of parental concern at age 5 was a significant predictor of late diagnosis, our result also demonstrated that even among the children who were diagnosed with ASD during secondary school, 75% had been identified by age 5 as having socio-behavioural difficulties. This proportion was significantly and substantially higher than that for those without a diagnosis of ASD throughout. This fits with findings from a clinical study showing that, compared to children diagnosed before age 5 years, children diagnosed later had higher cognitive ability. However, parental retrospective recall showed their autistic symptoms were already present at around age 4–5 and were only marginally less severe (Goodwin et al., 2018). This also fits with the finding that late-diagnosed autistic individuals consistently report having been identified as having adjustment difficulties long before these were understood in the context of ASD (Bargiela et al., 2016). Our result from the general population suggests that approaches aiming to encourage referral to a formal assessment at the first report of atypical social development by parents or teachers, and approaches targeting the primary school entry age could help promote earlier identification for those at risk of being diagnosed during school age.

Contrary to studies from the United States that find higher socio-economic status to be associated with earlier diagnosis (Bickel et al., 2015; Daniels & Mandell, 2014; Fountain et al., 2011; Mandell et al., 2005; Mazurek et al., 2014), studies in the United Kingdom have found lower socio-economic status to be associated with earlier diagnosis (Brett et al., 2016). However, in our study, there was a more complex, non-linear relationship between socio-economic status and timing of diagnosis: lower parental education was observed in both the earliest diagnosed and latest diagnosed children. Although the proportion of families living in the most health-deprived areas did not differ between diagnosis groups, fewer families in the earliest diagnosed group were living in relative household poverty. This result indicates that, although access to the healthcare system is universal in the United Kingdom, the process of receiving a diagnosis is still complex (Crane et al., 2016), with much reliance on parental navigation, which may create barriers particularly for those who are socially disadvantaged.

### Strengths and limitations

An important strength of our study was the relatively large group of children with ASD drawn from a population-representative UK cohort of children born at the beginning of the millennium and followed up to adolescence. We used multiple imputation to minimize data loss and survey weights to reduce the effect of attrition. The prospective longitudinal design and breadth of information available enabled us to examine multiple child, parent and teacher factors related to the timing of diagnosis with little recall bias.

There are also some limitations to our study. First, we relied on parental report to ascertain the timing of diagnosis which was not externally validated in the MCS. However, parental report of the child’s ASD diagnosis has been shown to have good reliability and has been used in previous population-based studies (Daniels & Mandell, 2014; Kogan et al., 2009; Miodovnik et al., 2015; Sheldrick et al., 2017). Relatedly, some children may lose or change their ASD diagnosis as they mature (Kogan et al., 2009). We were not able to explore the stability or current status of the parent-reported ASD diagnosis in the MCS because in the later sweeps (i.e. from the age 11 interview) the diagnostic status was asked only to those who did not report a prior diagnosis of ASD. However, excluding those whose parents reported losing an ASD diagnosis at the age 7 interview showed similar results. Second, we lacked information from direct assessment of the child including the severity of ASD symptoms which may have influenced the results (Fountain et al., 2011; Mandell et al., 2005; Mazurek et al., 2014; Sheldrick et al., 2017; Zwaigenbaum et al., 2019). Although our study variable regarding parent and teacher concerns over the child’s socio-behavioural difficulties may partly reflect the child’s severity, it would be important to include the severity of ASD symptoms obtained via direct assessment of the children in future studies. Third, our predictors were all measured at age 5 years, to explore predictors of timing of diagnosis at a crucial stage of childhood development, namely primary school entry. Future studies are needed to investigate earlier and later predictors of diagnostic timing. Fourth, aside from a parent-reported diagnosis of ADHD, information on preceding comorbid psychiatric diagnoses which may influence the timing of ASD diagnosis was not available to us. However, our sensitivity analysis showed that excluding children with ASD who had a preceding diagnosis for ADHD yielded relatively similar results. Future studies using linked clinical data with information on the age of ASD diagnosis and other psychiatric diagnoses may further ascertain determinants of the timing of diagnosis for ASD found in our study.

## Conclusion

Our findings warn healthcare and educational practitioners that high numbers of children, particularly those without cognitive delays and from poorer families, are likely to remain undiagnosed and therefore miss the opportunity for timely intervention and support for ASD before reaching adolescence. Actively linking schools and parents to facilitate dialogue can be the first step for these children. Offering information about relevant services (e.g. where and how to refer for consultation and assessment) may benefit parents, particularly those from a socially disadvantaged background. Given that many of the children with ASD had already been noticed as having socio-behavioural difficulties at age 5, strategies to promote earlier identification among school-aged children could target primary school entry age (i.e. age 5) and approaches to encourage referrals to a formal assessment at the first report of concerns over the child’s social development could be one way to address this. These approaches may benefit those children who would otherwise be diagnosed later.

## Supplemental Material

Supplementary_material – Supplemental material for Determinants of an autism spectrum disorder diagnosis in childhood and adolescence: Evidence from the UK Millennium Cohort Study Supplemental material, Supplementary_material for Determinants of an autism spectrum disorder diagnosis in childhood and adolescence: Evidence from the UK Millennium Cohort Study by Mariko Hosozawa, Amanda Sacker, William Mandy, Emily Midouhas, Eirini Flouri and Noriko Cable in Autism

## References

[bibr1-1362361320913671] American Psychiatric Association. (2013). Diagnostic and statistical manual of mental disorders (5th ed.).

[bibr2-1362361320913671] Baio J. Wiggins L. Christensen D. L. Maenner M. J. Daniels J. Warren Z. . . .Dowling N. F. (2018). Prevalence of autism spectrum disorder among children aged 8 years: Autism and Developmental Disabilities Monitoring Network, 11 sites, United States, 2014. Morbidity and Mortality Weekly Report Surveillance Summaries, 67(6), 1–23. 10.15585/mmwr.ss6706a1 PMC5919599 29701730

[bibr3-1362361320913671] Bargiela S. Steward R. Mandy W. (2016). The experiences of late-diagnosed women with autism spectrum conditions: An investigation of the female autism phenotype. Journal of Autism and Developmental Disorders, 46(10), 3281–3294. 10.1007/s10803-016-2872-8 27457364 PMC5040731

[bibr4-1362361320913671] Bickel J. Bridgemohan C. Sideridis G. Huntington N. (2015). Child and family characteristics associated with age of diagnosis of an autism spectrum disorder in a tertiary care setting. Journal of Developmental and Behavioral Pediatrics, 36(1), 1–7. 10.1097/DBP.0000000000000117 25539088

[bibr5-1362361320913671] Brett D. Warnell F. McConachie H. Parr J. R. (2016). Factors affecting age at ASD diagnosis in UK: No evidence that diagnosis age has decreased between 2004 and 2014. Journal of Autism and Developmental Disorders, 46(6), 1974–1984. 10.1007/s10803-016-2716-6 27032954 PMC4860193

[bibr6-1362361320913671] Corsello C. M. Akshoomoff N. Stahmer A. C. (2013). Diagnosis of autism spectrum disorders in 2-year-olds: A study of community practice. Journal of Child Psychology and Psychiatry, 54(2), 178–185. 10.1111/j.1469-7610.2012.02607.x 22905987 PMC3505251

[bibr7-1362361320913671] Crane L. Chester J. W. Goddard L. Henry L. A. Hill E. (2016). Experiences of autism diagnosis: A survey of over 1000 parents in the United Kingdom. Autism, 20(2), 153–162. 10.1177/1362361315573636 25810370

[bibr8-1362361320913671] Daniels A. M. Mandell D. S. (2014). Explaining differences in age at autism spectrum disorder diagnosis: A critical review. Autism, 18(5), 583–597. 10.1177/1362361313480277 23787411 PMC4775077

[bibr9-1362361320913671] Dawson G. (2008). Early behavioral intervention, brain plasticity, and the prevention of autism spectrum disorder. Development and Psychopathology, 20(3), 775–803. 10.1017/S0954579408000370 18606031

[bibr10-1362361320913671] Elliott C. Smith P. McCulloch K. (1997). British Ability Scales second edition (BAS II): Technical manual. National Foundation for Educational Research-Nelson.

[bibr11-1362361320913671] Fountain C. King M. D. Bearman P. S. (2011). Age of diagnosis for autism: Individual and community factors across 10 birth cohorts. Journal of Epidemiology and Community Health, 65(6), 503–510. 10.1136/jech.2009.104588 20974836 PMC3039707

[bibr12-1362361320913671] Goodman R. (1997). The Strengths and Difficulties Questionnaire: A research note. Journal of Child Psychology and Psychiatry, 38(5), 581–586. 9255702 10.1111/j.1469-7610.1997.tb01545.x

[bibr13-1362361320913671] Goodwin A. Matthews N. L. Smith C. J. (2018). Parent-reported early symptoms of autism spectrum disorder in children without intellectual disability who were diagnosed at school age. Autism, 23(3), 770–782. 10.1177/1362361318777243 29852752

[bibr14-1362361320913671] Hansen K . (2014). Millennium Cohort Study: A guide to the datasets (7th ed.). Centre for Longitudinal Studies, UCL Institute of Education, University College London.

[bibr15-1362361320913671] Hansen S. N. Schendel D. E. Parner E. T. (2015). Explaining the increase in the prevalence of autism spectrum disorders: The proportion attributable to changes in reporting practices. Journal of the American Medical Pediatrics, 169(1), 56–62. 10.1001/jamapediatrics.2014.1893 25365033

[bibr16-1362361320913671] Harel O. Mitchell E. M. Perkins N. J. Cole S. R. Tchetgen Tchetgen E. J. Sun B. Schisterman E. F. (2018). Multiple imputation for incomplete data in epidemiologic studies. American Journal of Epidemiology, 187(3), 576–584. 10.1093/aje/kwx349 29165547 PMC5860387

[bibr17-1362361320913671] Hull L. Petrides K. V. Allison C. Smith P. Baron-Cohen S. Lai M. C. Mandy W. (2017). Putting on my best normal: Social camouflaging in adults with autism spectrum conditions. Journal of Autism and Developmental Disorders, 47(8), 2519–2534. 10.1007/s10803-017-3166-5 28527095 PMC5509825

[bibr18-1362361320913671] Idring S. Lundberg M. Sturm H. Dalman C. Gumpert C. Rai D. . . .Magnusson C. (2015). Changes in prevalence of autism spectrum disorders in 2001-2011: Findings from the Stockholm youth cohort. Journal of Autism and Developmental Disorders, 45(6), 1766–1773. 10.1007/s10803-014-2336-y 25475364

[bibr19-1362361320913671] Johnson J. (2008). Millennium third survey follow-up: A guide to the school assessment datasets (1st ed.). Centre for Longitudinal Studies, Institute of Education.

[bibr20-1362361320913671] Klein D. (2014). MIMRGNS: Stata module to run margins after mi estimate. Department of Economics, Boston College. https://ideas.repec.org/c/boc/bocode/s457795.html

[bibr21-1362361320913671] Kogan M. D. Blumberg S. J. Schieve L. A. Boyle C. A. Perrin J. M. Ghandour R. M. . . .van Dyck P. C. (2009). Prevalence of parent-reported diagnosis of autism spectrum disorder among children in the US, 2007. Pediatrics, 124(5), 1395–1403. 10.1542/peds.2009-1522 19805460

[bibr22-1362361320913671] Lai M. C. Baron-Cohen S. (2015). Identifying the lost generation of adults with autism spectrum conditions. The Lancet Psychiatry, 2(11), 1013–1027. 10.1016/S2215-0366(15)00277-1 26544750

[bibr23-1362361320913671] Livingston L. A. Happe F. (2017). Conceptualising compensation in neurodevelopmental disorders: Reflections from autism spectrum disorder. Neuroscience & Biobehavioral Reviews, 80, 729–742. 10.1016/j.neubiorev.2017.06.005 28642070 PMC7374933

[bibr24-1362361320913671] Mandell D. S. Novak M. M. Zubritsky C. D. (2005). Factors associated with age of diagnosis among children with autism spectrum disorders. Pediatrics, 116(6), 1480–1486. 10.1542/peds.2005-0185 16322174 PMC2861294

[bibr25-1362361320913671] Mandy W. Pellicano L. St Pourcain B. Skuse D. Heron J. (2018). The development of autistic social traits across childhood and adolescence in males and females. Journal of Child Psychology and Psychiatry, 59(11), 1143–1151. 10.1111/jcpp.12913 29672866

[bibr26-1362361320913671] Mazurek M. O. Handen B. L. Wodka E. L. Nowinski L. Butter E. Engelhardt C. R. (2014). Age at first autism spectrum disorder diagnosis: The role of birth cohort, demographic factors, and clinical features. Journal of Developmental and Behavioral Pediatrics, 35(9), 561–569. 25211371 10.1097/DBP.0000000000000097

[bibr27-1362361320913671] Miodovnik A. Harstad E. Sideridis G. Huntington N. (2015). Timing of the diagnosis of attention-deficit/hyperactivity disorder and autism spectrum disorder. Pediatrics, 136(4), e830–837. 10.1542/peds.2015-1502 26371198

[bibr28-1362361320913671] National Initiative for Autism: Screening and Assessment. (2003). National Autism Plan for Children (NAPC): Plan for the identification, assessment, diagnosis and access to early interventions for pre-school and primary school aged children with autism spectrum disorders (ASD). The National Autistic Society for National Initiative for Autism: Screening and Assessment.

[bibr29-1362361320913671] National Institute for Health and Care Excellence. (2011, September). Autism spectrum disorder in under 19s: Recognition, referral and diagnosis [Clinical Guideline 128]. https://www.nice.org.uk/guidance/cg128 31999412

[bibr30-1362361320913671] Reed P. Osborne L. A. (2012). Diagnostic practice and its impacts on parental health and child behaviour problems in autism spectrum disorders. Archives of Disease in Childhood, 97(10), 927–931. 10.1136/archdischild-2012-301761 22806234

[bibr31-1362361320913671] Ruiz Calzada L. Pistrang N. Mandy W. P. (2012). High-functioning autism and Asperger’s disorder: Utility and meaning for families. Journal of Autism and Developmental Disorders, 42(2), 230–243. 10.1007/s10803-011-1238-5 21472359

[bibr32-1362361320913671] Shattuck P. T. Durkin M. Maenner M. Newschaffer C. Mandell D. S. Wiggins L. . . .Cuniff C. (2009). Timing of identification among children with an autism spectrum disorder: Findings from a population-based surveillance study. Journal of the American Academy of Child and Adolescent Psychiatry, 48(5), 474–483. 10.1097/CHI.0b013e31819b3848 19318992 PMC3188985

[bibr33-1362361320913671] Sheldrick R. C. Maye M. P. Carter A. S. (2017). Age at first identification of autism spectrum disorder: An analysis of two US surveys. Journal of the American Academy of Child and Adolescent Psychiatry, 56(4), 313–320. 10.1016/j.jaac.2017.01.012 28335875 PMC5367515

[bibr34-1362361320913671] Steiner A. M. Goldsmith T. R. Snow A. V. Chawarska K. (2012). Practitioner’s guide to assessment of autism spectrum disorders in infants and toddlers. Journal of Autism and Developmental Disorders, 42(6), 1183–1196. 10.1007/s10803-011-1376-9 22057879 PMC4878115

[bibr35-1362361320913671] White I. R. Royston P. Wood A. M. (2011). Multiple imputation using chained equations: Issues and guidance for practice. Statistics in Medicine, 30(4), 377–399. 10.1002/sim.4067 21225900

[bibr36-1362361320913671] Williams E. Thomas K. Sidebotham H. Emond A. (2008). Prevalence and characteristics of autistic spectrum disorders in the ALSPAC cohort. Developmental Medicine & Child Neurology, 50(9), 672–677. 10.1111/j.1469-8749.2008.03042.x 18754916

[bibr37-1362361320913671] Zuckerman K. Lindly O. J. Chavez A. E. (2017). Timeliness of autism spectrum disorder diagnosis and use of services among U.S. elementary school-aged children. Psychiatric Services, 68(1), 33–40. 10.1176/appi.ps.201500549 27476809 PMC5205571

[bibr38-1362361320913671] Zwaigenbaum L. Duku E. Fombonne E. Szatmari P. Smith I. M. Bryson S. E. . . .Bruno R. (2019). Developmental functioning and symptom severity influence age of diagnosis in Canadian preschool children with autism. Paediatrics & Child Health, 24(1), e57–e65. 10.1093/pch/pxy076 30906197 PMC6376294

