# Supplementary material for: Determinants of an autism spectrum disorder diagnosis in childhood and adolescence: Evidence from the UK Millennium Cohort Study
Source: Autism. 2020 May 5;24(6):1557–65. doi: 10.1177/1362361320913671 (PMC7645602; doi:10.1177/1362361320913671)
Supplement: Supplementary_material – Supplemental material for Determinants of an autism spectrum disorder diagnosis in childhood and adolescence: Evidence from the UK Millennium Cohort Study [file Supplementary_material.pdf]

Supplementary Table S1

Characteristics of the MCS sample by the timing of diagnosis for autism spectrum disorder

	Total MCS sample at age 5 interview (N = 15,431)[a]							
	ASD (n = 581)						No diagnosis of ASD by age 14 (n = 14,850)	
	Before school (n = 126)		During primary school (n = 300)		During secondary school (n = 155)			
	n	%	n	%	n	%	n	%
Sex of the child								
Male	104	80.6	233	76.9	111	73.3	7,424	49.9
Female	22	19.4	67	23.1	44	26.7	7,426	50.1
Cognitive ability at age 5								
Within normal range	63	87.5	235	86.4	136	93.1	13,928	96.3
Below 1 SD	11	12.5	41	13.6	13	6.9	622	3.8
Parental highest education								
A-level or above	63	46.0	188	63.4	80	51.1	8,978	61.7
Below A-level	63	54.0	111	36.6	75	48.9	5,861	38.3
Low household income								
Yes	42	31.1	119	37.3	65	45.5	4,977	30.3
No	84	68.9	181	62.7	90	54.5	9,787	69.7
Neighbourhood health deprivation								
Yes	12	7.5	36	10.6	27	15.2	1,922	10.7
No	114	92.5	264	89.5	128	84.8	12,927	89.3
Parent-reported diagnosis of ADHD by age 14								
Yes	41	31.8	116	37.8	39	24.4	295	2.0
No	85	68.3	184	62.2	116	75.6	14,553	98.0
Preceding parent-reported diagnosis of ADHD[b]								
Yes	-	-	42	14.7	18	12.2	-	-
No	-	-	258	85.3	137	87.8	-	-

Note. Unweighted numbers and weighted percentages are shown. N varies due to missing data. [a] 28 children without a valid answer for diagnosis of ASD at all waves are excluded. [b] Defined as parents reporting a diagnosis of ADHD in waves prior to reporting a diagnosis of ASD.

Supplementary Table S2

The observed prevalence of parental/teacher concerns over the child's socio-behavioural difficulties

	Total MCS sample at age 5 interview (N = 15,431) [a]							
	ASD (n = 581)							
	Before school (n = 126)		During primary school (n = 300)		During secondary school (n = 155)		No diagnosis of ASD by age 14 (n = 14,850)	
	n	%	n	%	n	%	n	%
Having parental concerns over the child's *socio-behavioural* difficulties at age 5								
Yes	103	90.9	217	71.9	88	61.6	3,666	25.4
No	15	9.1	77	28.1	58	38.4	10,566	74.6
Teacher evaluated social developmental delay at age 5								
Yes	55	66.5	108	42.0	52	51.3	2,024	16.7
No	27	33.5	127	58.0	61	48.6	9,546	83.4
Having parental concerns over the child's *social* difficulties at age 5[b]								
Yes	97	84.5	155	50.8	71	46.2	3,698	24.9
No	22	15.5	142	49.2	78	53.8	10,706	75.1

Note. Unweighted numbers and weighted percentages are shown. N varies due to missing data. [a]28 children without a valid answer for diagnosis of ASD at all waves are excluded.[b]Scoring either above 1SD on the peer subscale or below 1SD on the prosocial subscale of the parent-reported Strengths and Difficulties Questionnaire measured at age 5.

Supplementary Table S3

Sensitivity analysis for determinants of the timing of diagnosis for autism spectrum disorder

	Timing of diagnosis for autism spectrum disorder	
	During primary school[a]	During secondary school[a]
Female sex	0.90 (0.47 to 1.73)	1.11 (0.53 to 2.32)
Cognitive delay at age 5	1.23 (0.53 to 2.86)[b]	0.53 (0.20 to 1.36)[b]
Low parental education (below A-level)	0.42 (0.24 to 0.74)	0.66 (0.37 to 1.18)
Low household income	2.21 (1.19 to 4.09)	2.53 (1.24 to 5.19)
Having parental concerns over the child's *social* difficulties at age 5	0.20 (0.11 to 0.39)	0.17 (0.08 to 0.33)
Teacher evaluated social developmental delay	0.53 (0.29 to 0.95)	0.75 (0.39 to 1.45)
Neighbourhood health deprivation	1.46 (0.75 to 2.84)	1.69 (0.69 to 4.15)

Note. Adjusted Relative risk ratios (95% confidence intervals) are reported. [a]The Before school group is taken as reference. [b]Significant difference between During primary school group and During secondary school group. All analyses adjusted for multiple birth indicator.

Supplementary Table S4

Sensitivity analysis for determinants of the timing of diagnosis for autism spectrum disorder without children with preceding diagnosis of attention-deficit/hyperactivity disorder (ADHD)

	Timing of diagnosis for autism spectrum disorder	
	During primary school[a]	During secondary school[a]
Female sex	1.12 (0.57 to 2.17)	1.33 (0.66 to 2.71)
Cognitive delay	1.10 (0.49 to 2.48)[b]	0.43 (0.15 to 1.18)[b]
Low parental education (below A-level)	0.42 (0.24 to 0.74)	0.61 (0.34 to 1.09)
Low household income	1.76 (0.93 to 3.31)	2.19 (1.07 to 4.46)
Having parental concerns over the child's socio-behavioural difficulties at age 5	0.28 (0.12 to 0.61)	0.17 (0.08 to 0.39)
Teacher evaluated social developmental delay	0.47 (0.26 to 0.85)	0.70 (0.36 to 1.37)
Neighbourhood health deprivation	1.77 (0.86 to 3.64)	1.93 (0.66 to 5.69)

Note. Adjusted Relative risk ratios (95% confidence intervals) are reported. [a]The Before school group is taken as reference. [b]Significant difference between During primary school group and During secondary school group. All analyses adjusted for multiple birth indicator.

Supplementary Table S5

Sensitivity analysis for determinants of the timing of diagnosis for autism spectrum disorder without 15 children who lost their parent-reported diagnosis at age 7

	Timing of diagnosis for autism spectrum disorder	
	During primary school[a]	During secondary school[a]
Female sex	0.98 (0.48 to 2.01)	1.19 (0.56 to 2.50)
Cognitive delay	1.10 (0.48 to 2.53)[b]	0.48 (0.18 to 1.31)[b]
Low parental education (below A-level)	0.43 (0.24 to 0.79)	0.69 (0.39 to 1.25)
Low household income	2.24 (1.16 to 4.35)	2.56 (1.19 to 5.52)
Having parental concerns over the child's socio-behavioural difficulties at age 5	0.19 (0.07 to 0.54)[b]	0.11 (0.04 to 0.33)[b]
Teacher evaluated social developmental delay	0.40 (0.22 to 0.75)	0.60 (0.30 to 1.17)
Neighbourhood health deprivation	1.63 (0.77 to 3.41)	1.88 (0.64 to 5.54)

Note. Adjusted Relative risk ratios (95% confidence intervals) are reported. [a]The Before school group is taken as reference. [b]Significant difference between During primary school group and During secondary school group. All analyses adjusted for multiple birth indicator.
